# Inclusion of IJOTM in Scopus and EMBASE

**Published:** 2012-05-01

**Authors:** Karim Vessal

It was a great pleasure to realize that the *International Journal for Organ Transplantation Medicine (IJOTM)* has been incorporated in the respected Elsevier databases, *Scopus* and *EMBASE/Excerpta Medica*. The editorial staff of the *IJOTM* acknowledges this highly esteemed recognition to the sincere support of the Avicenna Transplantation Center and the continuous encouragement of *Shiras University of Medical Sciences*.

The Journal is the official publication of the Iranian Society of Organ Transplantation and also the Transplantation Division of the Avicenna Medical Institutions which boasts a brilliant record in patient throughput on international scale as well as a remarkable pace of growth in scientific advancement over the past decade.

Thanks to the steady efforts of a large dedicated team of physicians and scientists, the center has recently launched on expansion of its infrastructure as well as the diversity of organs transplanted. It is anticipated that the organization will stand out as one of the leading centers of transplantation medicine in the near future.

Shiraz, the city which harbors the Avicenna Institutes, enjoys an outstanding reputation as a medical center throughout the Persian history. Following the downfall of the renowned Medical Academy of Jundishapur, which was the first teaching center of medicine in the world, many of its erudites settled in nearby Shiraz to proceed with the advancement of science and medicine and thus create centers of their own. This led to the emergence of numerous scholars and polymaths who have influenced the development of medicine in the world. It is the aspiration of Avicenna Medical Institutions to be capable of reviving the past scientific glory of the country and pass its heritage of excellence on to the future generations.

